# Exploring self-esteem and personality traits as predictors of mental wellbeing among Chinese university students: the mediating and moderating role of resilience

**DOI:** 10.3389/fpsyg.2024.1308863

**Published:** 2024-04-10

**Authors:** Zhenti Cui, Zihan Lin, Jingjie Ren, Yingdong Cao, Xiaofei Tian

**Affiliations:** ^1^School of Medicine, Sias University, Xinzheng, China; ^2^The Affiliated Cancer Hospital of Zhengzhou University & Henan Cancer Hospital, Zhengzhou, China; ^3^The Seventh Affiliated Hospital of Sun Yat-sen University, Shenzhen, China

**Keywords:** self-esteem, personality, resilience, mental wellbeing, college students

## Abstract

**Introduction:**

The mental health of university students is influenced by a variety of factors, including self-esteem and personality traits, with resilience playing a crucial role in mediating and moderating these relationships. This study investigates the intricate dynamics affecting mental well-being in Chinese university students, focusing on the roles of self-esteem, personality traits, and the interventional effects of resilience.

**Methods:**

A cross-sectional survey was conducted with 689 students, aged on average 20.3 years, between April and July 2022. The Warwick-Edinburgh Mental Wellbeing Scale (WEMWBS) was used to assess mental well-being, while resilience, personality traits, and self-esteem were evaluated using the revised Connor-Davidson Resilience Scale (CD-RISC), the Chinese version of the Big Five Inventory-2 (BFI-2), and the Texas Social Behavior Inventory (TSBI), respectively.

**Results:**

Analysis revealed significant correlations between self-esteem, personality traits, and both resilience and mental well-being. Resilience was found to partially mediate the relationship between self-esteem and mental well-being and fully mediate certain aspects of the relationship between personality traits and mental well-being. Additionally, tenacity and autonomy were identified as moderators in the link between specific personality traits and mental well-being.

**Discussion:**

The findings highlight the complex interplay between self-esteem, personality traits, resilience, and mental well-being, underscoring the critical role of resilience. This insight is pivotal for developing targeted interventions to bolster mental well-being among university students, emphasizing the need for multifaceted support strategies to enhance student mental health.

## 1 Introduction

### 1.1 Self-esteem and personality traits and its relationship with mental wellbeing

Within the realm of mental health and wellbeing, the interplay between self-esteem, personality traits, and mental wellbeing emerges as a critical avenue for investigation. Self-esteem, a multifaceted self-assessment encompassing a sense of personal value and significance, holds considerable importance in individual development (Rosenberg, 1979). This inner mindset, shaped by individuals themselves, plays a pivotal role in their ability to navigate everyday stressors, maintain healthy social interactions, and foster physical wellbeing (Doré, 2017). Extensive research has explored the impact of self-esteem on psychological and physiological states, revealing its association with emotional and physical health outcomes (Tsaousis, 2016). High self-esteem has been linked to resilience against negative influences, whereas low self-esteem has been connected to difficulties in coping with stressors and potential negative outcomes (Gao et al., 2019).

Mental wellbeing, defined as a state of positive functioning where individuals recognize their capabilities, effectively manage stressors, contribute to their communities, and experience overall life satisfaction (World Health Organization, 2004), has been consistently linked to self-esteem. This association is evidenced by studies indicating that individuals with higher levels of self-esteem reported greater happiness and better mental wellbeing (Baumeister et al., 2003). Conversely, lower self-esteem has been associated with increased anxiety and defensive behaviors (Pyszczynski et al., 2004). Recent inquiries into the relationship between anxiety and self-esteem have demonstrated that enhancing self-esteem can help alleviate stress and anxiety-related issues (Galanakis et al., 2016). Moreover, among college students, the association between self-esteem and mental wellbeing has been investigated extensively, with findings suggesting that self-esteem and mental wellbeing are closely linked (Gao et al., 2015). However, a nuanced exploration of the pathways through which self-esteem affects mental wellbeing remains underexplored.

### 1.2 Resilience and its role in mental wellbeing

Adding depth to this context is the concept of resilience, which encompasses an individual’s capacity to adapt to disturbances and swiftly return to a state of equilibrium (Bonanno, 2004). Research investigating the connection between resilience and mental wellbeing has yielded significant insights. Enhanced individual resilience has been consistently associated with improved mental wellbeing (Ghanei Gheshlagh et al., 2017). This relationship has prompted the development of preventive interventions, such as cognitive behavioral therapy, that aim to bolster mental wellbeing by enhancing resilience (Joyce et al., 2018).

Further exploration of the interplay between self-esteem, resilience, and mental wellbeing is particularly relevant for young adults, a demographic vulnerable to the adverse effects of various stressors (Kannarkat et al., 2020; Wu et al., 2022). This transitionary phase of life is marked by profound shifts in social interactions and self-perception (Skowron et al., 2008). Individuals’ self-esteem undergoes fluctuations in response to evolving societal experiences, potentially influencing their mental wellbeing (Chung et al., 2014). Similarly, while resilience can remain relatively stable throughout childhood, its dynamics may undergo significant changes during young adulthood (Pargas et al., 2010).

The exploration of personality traits and their impact on mental wellbeing has garnered increasing attention, shedding light on potential underlying mechanisms. Wiebe et al. (2018) summarized various theoretical explanations for the relationship between personality and health. Among these, the transactional stress-moderation model posits that personality influences the frequency and intensity of stress-induced physiological responses, ultimately affecting health outcomes (Wiebe et al., 2018). Other perspectives, such as health and risk behaviors are shaped by processes that are hypothesized within this framework, whereas illness behavior and self-regulation are informed by a broader conceptual understanding, and biological and early experience conceptual frameworks have also provided unique insights into the interface between personality and health.

### 1.3 Research rationale

The impetus for concentrating on college students in our study is rooted in the unique developmental challenges and transitions characteristic of this life stage, which can significantly influence mental wellbeing. The college years are a period of profound change, where students navigate academic pressures, evolving social relationships, and the task of defining their identities and future paths (Zhang, 2022). These challenges underscore the importance of examining mental wellbeing within this demographic, as the way students cope with these stressors can have lasting effects on their psychological health.

A key motivation for focusing specifically on the impact of personal traits, such as self-esteem and personality dimensions, is their critical role in shaping students’ responses to the myriad stressors encountered during college (Theron et al., 2022). These personal traits influence how students perceive, interpret, and navigate their environments, directly impacting their mental wellbeing. The exploration of these traits is particularly pertinent in the context of Chinese higher education, where cultural and educational pressures might uniquely interact with personal traits to influence students’ mental health.

Despite existing research on the role of self-esteem and personality traits in mental wellbeing, there remains a significant gap in our understanding of these dynamics within the specific setting of Chinese higher education. This gap is especially pronounced considering the unique socio-cultural and educational pressures faced by this population, which may affect the development and expression of resilience and, in turn, mental wellbeing (Bag et al., 2022).

This study seeks to address these gaps by not only examining the direct effects of self-esteem and personality traits on mental wellbeing but also exploring the nuanced ways in which resilience may mediate and moderate these relationships among Chinese university students. By investigating these interactions, we aim to identify potential avenues for targeted interventions designed to bolster student mental health and resilience, thereby better equipping them to handle the academic and social challenges of college life.

### 1.4 Theoretical framework

Our study is grounded in an integrated theoretical framework that incorporates socioemotional selectivity theory, self-determination theory, and resilience theory to examine the mental wellbeing of Chinese university students.

Socioemotional selectivity theory provides valuable insights into how individuals prioritize their goals based on their perceptions of time, a concept particularly relevant to university students who are often at a critical juncture of planning their futures while navigating the immediate demands of their academic and personal lives (Löckenhoff and Carstensen, 2004). In the Chinese higher education context, where there is a strong emphasis on future success and career achievement, this theory helps explain how such future-oriented perspectives might impact students’ social connections and emotional wellbeing.

Self-determination theory emphasizes the importance of meeting basic psychological needs—autonomy, competence, and relatedness—for optimal wellbeing (Deci and Ryan, 2000). This theory is particularly pertinent in the educational setting, where the fulfillment of these needs can be significantly impacted by academic structures and social environments. For Chinese university students, the pressure to conform to high academic standards and societal expectations may challenge their sense of autonomy and competence, while the competitive environment may affect their sense of connectedness.

Resilience theory focuses on individuals’ ability to withstand and adapt to life stressors and adversity (Van Breda, 2001). This theory is particularly relevant for understanding how university students manage the unique challenges and pressures they face, and how resilience can serve both as a protective factor and as a mechanism of recovery that mediates and moderates the effects of personality traits and self-esteem on their mental wellbeing.

By integrating these three theoretical perspectives, our framework seeks to capture the dynamic interplay between students’ personal traits, their perceived ability to meet psychological needs, and their resilience in shaping mental wellbeing. This approach allows for understanding how internal traits and external stressors interact to shape the mental wellbeing of university students, providing a nuanced understanding of the factors that contribute to their psychological health and paving the way for targeted interventions. Thus, based on the theoretical framework, our hypothesizes are listed as follows:

(a) Self-esteem and personality traits (as defined by the Big Five personality dimensions) have significant predictive roles in determining the mental wellbeing of Chinese university students.
(b) Resilience will serve as a mediating factor in this relationship, wherein higher levels of resilience are expected to strengthen the positive impact of self-esteem and adaptive personality traits on mental wellbeing.
(c) The interaction between resilience and specific personality traits may exhibit a moderating effect on mental wellbeing, suggesting a more nuanced interplay between these variables.

### 1.5 Purpose of the study

Given the complexity of the associations between self-esteem, personality traits, resilience, and mental wellbeing, there is a compelling need to unravel the nuanced pathways that underlie these relationships. While some research has explored certain aspects of these connections, critical gaps remain unaddressed. Notably, few studies have thoroughly investigated the pathway from self-esteem to mental wellbeing while considering the mediating and moderating role of resilience. Prior work has underscored the potential importance of factors like empathic tendencies and geographical context in shaping this relationship (Gao et al., 2015). Although the longitudinal study by Marshall et al. (2015) partially examined the impact of self-esteem on mental wellbeing, the mediating and moderating influence of resilience remained unexplored.

Thus, this study aims to bridge existing gaps in the literature by investigating the mediation and moderation roles of resilience and its sub-dimensions in the context of Chinese college students’ mental wellbeing. Specifically, our objectives are:

(a) To explore how self-esteem and personality traits predict mental wellbeing among Chinese university students.
(b) To examine the extent to which resilience mediates the relationship between self-esteem, personality traits, and mental wellbeing.
(c) To investigate whether resilience, along with its sub-dimensions, moderates the impact of self-esteem and personality traits on mental wellbeing.

## 2 Materials and methods

### 2.1 Study design and participants

This research was conducted as a pilot study employing a cross-sectional design from April to July 2022. Given its exploratory nature aimed at investigating the interplay between self-esteem, personality traits, resilience, and mental wellbeing, the study was not predicated on *a priori* sample size calculations. Instead, the focus was on generating preliminary insights that could inform future, more comprehensive research.

Participants were college students selected from Henan province, a region chosen for its diverse and densely populated characteristics, reflective of a broad segment of China’s student population. The province’s central location and the mix of urban and rural areas provide a unique context for exploring student mental wellbeing. The sampling involved a two-stage random selection: initially picking one city from each of the five key regions (North, West, East, South, and Central Henan) followed by the selection of one or two universities within each city. This stratified approach aimed to enhance the diversity of the participant pool within the logistical constraints of the study.

An online survey was administered using Wenjuanxing, a widely used online survey platform in China known for its robust features suitable for academic research.^1^ Firstly, we carefully designed the questionnaire on this platform with a combination of single-choice and Likert scale questions to effectively capture data on self-esteem, personality traits, resilience, and mental wellbeing. Then, the online survey was distributed by the research assistant who have the social connection with the university selected. Each assistant was responsible for specific universities and used established WeChat groups within these institutions to reach potential participants. This method ensured targeted and efficient access to our desired demographic of Chinese university students.

The study was conducted in accordance with the Declaration of Helsinki and approved by the Ethics Committee of the Sias University, and the local schools that participated (project number: LL2022-0315).

### 2.2 Measures

Four distinct measurement instruments were employed to assess each construct in this study. To gauge resilience, the revised Chinese version of the Connor-Davidson Resilience Scale (CD-RISC) was utilized, adapted for the cultural context of Chinese college students (Chen et al., 2013). The revised CD-RISC comprises 19 items distributed across three sub-scales: adaptability, tenacity, and autonomy. Respondents rated the extent to which each item applied to them on a 5-point Likert scale. The scale’s total score, ranging from 0 to 76, offers insight into an individual’s level of resilience. The revised CD-RISC has demonstrated strong reliability and validity, with a Cronbach’s alpha of 0.937 for this study. The revised CD-RISC has shown reliability and validity for assessing resilience in Chinese college students (Chen et al., 2013).

Self-esteem was assessed using the Texas Social Behavior Inventory (TSBI), a well-established instrument for evaluating self-esteem (Helmreich and Stapp, 1974). The TSBI, featuring 32 items, prompts participants to rate the extent to which each statement aligns with their self-perception on a 5-point Likert scale. Some items are reverse scored to mitigate response bias. The total TSBI score, ranging from 0 to 128, provides an indication of participants’ self-esteem levels. The reliability and validity of the TSBI have been previously established, with a Cronbach’s alpha of 0.849 for this study.

Personality traits were evaluated using the Chinese version of the Big Five Inventory–2 (BFI-2), which assesses five domains: extraversion, agreeableness, conscientiousness, negative emotionality, and open-mindedness (Soto and John, 2017). Participants rated the extent to which each item described them on a 5-point scale. The scores for each domain were computed by averaging the respective items, generating a comprehensive assessment of participants’ personality traits. The BFI-2 has been demonstrated to possess good reliability and validity across its domains in this Chinese population (Zhang et al., 2021). The Cronbach’s alpha reliability scores for the BFI-2 were notably high, with open-mindedness at 0.84, conscientiousness at 0.87, extraversion at 0.86, agreeableness at 0.81, and negative emotionality at 0.88.

The Warwick-Edinburgh Mental Wellbeing Scale (WEMWBS) was employed to measure mental wellbeing (Tennant et al., 2007). Consisting of 14 items, participants rated the frequency of positive emotional experiences they had over a specified period on a 5-point scale. Scores ranging from 14 to 70 provide an indication of an individual’s mental wellbeing level. The WEMWBS has been validated for use in both student and general populations, particularly among university students, with strong reliability (Cronbach’s alpha = 0.939 for this study) (Fung, 2019).

### 2.3 Statistical analysis

All data were securely stored online using encryption to ensure data integrity and privacy. Statistical analyses were performed using R and R Studio Version 2022.07.0+548. The process involved a sequence of steps to address the research questions and hypotheses. Any incomplete or missing responses were handled through imputation methods (e.g., mean imputation or multiple imputation) to ensure that the available data remained complete for analysis. Additionally, sensitivity analysis was conducted to assess the impact of missing data on the results before processing further analysis. The results of the sensitivity analysis revealed that the findings remained consistent and robust, suggesting that missing data did not significantly influence the overall results of the study.

**FIGURE 1:**
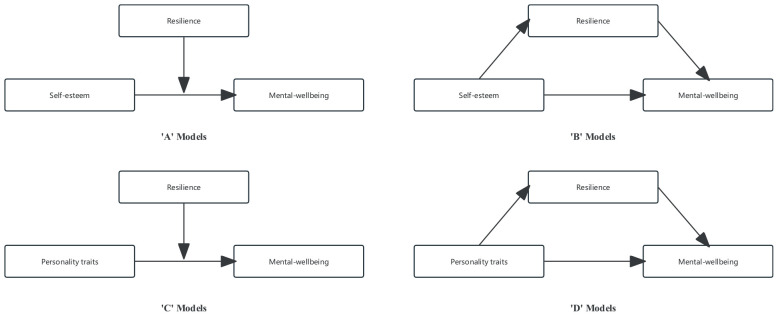
The theoretical mediating and moderating models in this study. All models included three sub-models according to different type of resilience. Both ‘C’ and ‘D’ models included five sub-models according to different traits of personality; ‘A’ and ‘C’ models were employed to describe the moderating effect of resilience on the association between self-esteem, personality traits and mental wellbeing. ‘B’ and ‘D’ models were used to test the mediating effect of resilience.

Descriptive analyses were initially conducted to provide an overview of the study’s participants. For continuous variables, mean (M) ± standard deviation (SD) was used, while categorical variables were summarized using frequencies and percentages. To present the model of mediation and moderation analysis, Figure 1 was prepared to illustrate the sequence of steps involved.

Following the descriptive analysis, correlation and reliability analyses were performed to examine the relationships among the study’s variables. Pearson correlation coefficients were computed to determine the associations between self-esteem, personality traits, resilience, and mental wellbeing. The Cronbach’s alpha coefficient was calculated to assess the internal consistency of the measurement scales.

Mediation analyses were carried out in four sequential steps using regression analysis, as outlined by Kenny (2024). In Step 1, the relationship between the predictor (self-esteem or personality traits) and the dependent variable (mental wellbeing) was examined. Step 2 involved testing the association between the predictor and the mediator (resilience or its sub-dimensions) using regression. Step 3 encompassed a multivariate regression analysis, where mental wellbeing was regressed on the predictor (self-esteem or personality traits) and the mediator (resilience or its sub-dimensions). In Step 4, the mediation effect was evaluated by analyzing the results of the regression model from Step 3. The size of the effect and its 95% confidence interval (95% CI) were presented. For mediation analysis, we utilized the mediate() function from the mediation package in R, designed specifically for such causal mediation analyses.

For the moderation analysis, interaction terms were introduced to assess the potential moderating roles of resilience and its sub-dimensions. A four-step process was adopted (Frazier et al., 2004), starting with a control model that included demographic variables (sex and age) in Step 1. Step 2 incorporated the predictor (self-esteem or personality traits) to create Model 2. Resilience and its sub-dimensions were added as moderator variables in Step 3 to create Model 3. In Step 4, interaction terms were introduced into Model 3 to create Model 4. Changes in the R-squared values associated with the interaction terms determined the moderation effects. All models were adjusted by age and sex. For the creation of moderation models, the lm() function was employed, and the ggpredict() function from the ggeffects package was used to visualize the interactions within these models.

All statistical tests were two-sided, and the significance level was set at alpha = 0.05. These analytical steps allowed for the systematic exploration of the relationships between self-esteem, personality traits, resilience, and mental wellbeing, enhancing the robustness of the study’s findings.

## 3 Results

### 3.1 Demographic information

Participants’ characteristics are shown in Table 1. The mean age of the participants was 20.3 years (SD = 1.4), with a majority being female (55.3%), of Han ethnicity (98.1%), and from urban areas (59.8%). In total, 7% of participants were from single parent, 15% consumed alcohol, 3.5% consumed cigarettes, and 6.7% had physical illness. The education level of most of students’ fathers (541, 78.5%) and mothers (554, 80.4%) was less then junior college.

**TABLE 1:** Demographic information.

Variables		No (%)
Gender	Male	308 (44.7)
	Female	381 (55.3)
Ethnicity	Han	676 (98.1)
	Minorities	13 (1.9)
Inhabitation	Rural	412 (59.8)
	Urban	277 (40.2)
Single parent	Yes	48 (7)
	No	641 (93)
Drinking	Yes	103 (15)
	No	586 (85.1)
Smoking	Yes	24 (3.5)
	No	665 (96.5)
Physical illness	Yes	46 (6.7)
	No	643 (93.3)
Chronic constipation	Yes	55 (8)
	No	634 (92)
Paternal education	High school and below	541 (78.5)
	Junior college	77 (11.2)
	Bachelor	68 (9.9)
	Master	2 (0.3)
	PhD	1 (0.1)
Maternal education	High school and below	554 (80.4)
	Junior college	79 (11.5)
	Bachelor	54 (7.8)
	Master	1 (0.1)
	PhD	1 (0.1)
Total		689 (100)

### 3.2 Correlations

The initial exploration of the data revealed significant correlations between the variables under study. All variables exhibited positive and significant correlations with each other, with the exception of negative emotionality, which showed negative correlations with all other variables (*r* = −0.328 to −0.573, *p* < 0.001). Table 2 provides detailed information on these correlation results.

**TABLE 2:** Correlation of scales and subscales.

Variables	2	3	4	5	6	7	8	9	10
Self-esteem (1)	0.60***	0.58***	0.56***	0.53***	0.70***	0.11***	0.27***	−0.40***	0.34***
Mental wellbeing (2)	1	0.77***	0.73***	0.70***	0.55***	0.34***	0.43***	−0.57***	0.40***
Adaptability (3)		1	0.78***	0.76***	0.49***	0.34***	0.56***	−0.51***	0.45***
Tenacity (4)			1	0.68***	0.50***	0.32***	0.48***	−0.53***	0.40***
Autonomy (5)				1	0.45***	0.25***	0.40***	−0.51***	0.36***
Extraversion (6)					1	0.19***	0.30***	−0.41***	0.38***
Agreeableness (7)						1	0.54***	−0.38***	0.29***
Conscientiousness (8)							1	−0.45***	0.38***
Negative emotionality (9)								1	−0.33***
Open-mindedness (10)									1

***p < 0.001 (two-tailed). “Self-esteem” was measured using the Texas Social Behavior Inventory (TSBI); “Mental wellbeing” was assessed by the Warwick-Edinburgh Mental Wellbeing Scale (WEMWBS); “Adaptability” and “Tenacity” and “Autonomy” were subscales of the Connor-Davidson Resilience Scale (CD-RISC); “Extraversion,” “Agreeableness,” “Conscientiousness,” “Negative emotionality” and “Open-mindedness” personality traits were evaluated by Chinese version of the Big Five Inventory–2 (BFI-2).

### 3.3 Mediation analysis of resilience with self-esteem as predictor

Mediation analysis was conducted to examine the potential mediating role of resilience in the relationship between self-esteem and mental wellbeing. The results are summarized in Table 3. The total effect of self-esteem on mental wellbeing was 0.43 (95% CI = 0.38 to 0.47, *p* < 0.001). After introducing resilience into the model, the direct effect of self-esteem on mental wellbeing decreased (ACME = 0.22 to 0.28, *p* < 0.001). Resilience explained a significant proportion of the variance in mental wellbeing when self-esteem was used as a predictor [Mediation (%) = 51.6 to 66.76].

**TABLE 3:** The mediation role of resilience in the association between self-esteem and mental wellbeing (‘B’ model).

Statistic outcome	Mediator		
	**Adaptability**	**Tenacity**	**Autonomy**
Total effect	0.43 (0.38, 0.47)		
Direct effect (ADE)	0.14 (0.1, 0.18)	0.2 (0.16, 0.25)	0.21 (0.17, 0.25)
Mediated effect (ACME)	0.28 (0.24, 0.33)	0.22 (0.18, 0.26)	0.22 (0.18, 0.26)
Mediation (%)	66.76 (59.17, 76)	52.1 (43.8, 61)	51.6 (43.5, 59)
Partial mediation	Yes	Yes	Yes
Full mediation	No	No	No

ADE, average direct effect; ACME, average causal mediated effect.

### 3.4 Mediation analysis of resilience with personality traits as predictor

Table 4 illustrates the mediation effect of resilience in the association between five personality traits and mental wellbeing. The total effect of personality traits on mental wellbeing ranges between 0.52 and 0.81, while only negative emotionality personality traits was negative associated with mental wellbeing [β = −0.81 (−0.89, −0.73), *p* < 0.001]. The effect of any personality traits on mental wellbeing decreased after adding any type of resilience as mediator, while the effect of conscientiousness and open mindedness on mental wellbeing with adaptability as mediator were insignificant [β = 0.01 (−0.07, 0.08), *p* > 0.05 for conscientiousness with resilience as mediator; β = 0.01 (−0.08, 0.08), *p* > 0.05 for conscientiousness; β = 0.04 (−0.04, 0.12), *p* > 0.05 for open mindedness]. In this model, resilience accounts for around or more than half of variance in mental wellbeing with five personality traits as predictor. Adaptability fully mediated the relationship between conscientiousness and open-mindedness on mental wellbeing. The proportion of mediation was 99.2% (88.2%, 113%) and 93% (81.3%, 107%), respectively.

**TABLE 4:** The mediation role of resilience in the association between self-esteem and mental wellbeing (‘D’ model).

Statistic outcome	Independent variable				
	**Extraversion**	**Agreeableness**	**Conscientiousness**	**Negative emotionality**	**Open mindedness**
Total effect	0.85 (0.77, 0.95)	0.52 (0.41, 0.63)	0.66 (0.57, 0.75)	−0.81 (−0.89, −0.73)	0.62 (0.5, 0.74)
**Adaptability as mediator**					
Direct effect (ADE)	0.31 (0.23, 0.38)	0.11 (0.04, 0.19)	0.01 (−0.08, 0.08)	−0.3 (−0.37, −0.22)	0.04 (−0.04, 0.12)
Mediated effect (ACME)	0.55 (0.46, 0.64)	0.41 (0.31, 0.5)	0.66 (0.58, 0.76)	−0.51 (−0.6, −0.44)	0.57 (0.48, 0.67)
Mediation (%)	64.3 (56.3, 72)	78.08 (66.63, 91)	**99.236 (88.154, 113)**	63.1 (55.2, 71)	**93.03 (81.34, 107)**
Partial mediation	Yes	Yes	No	Yes	No
Full mediation	No	No	Yes	No	Yes
**Tenacity as mediator**					
Direct effect (ADE)	0.38 (0.3, 0.47)	0.17 (0.08, 0.25)	0.18 (0.09, 0.26)	−0.34 (−0.42, −0.25)	0.18 (0.09, 0.27)
Mediated effect (ACME)	0.47 (0.4, 0.56)	0.35 (0.27, 0.44)	0.49 (0.42, 0.57)	−0.48 (−0.56, −0.41)	0.44 (0.36, 0.53)
Mediation (%)	55.3 (47.3, 64)	67.7 (55.87, 82)	73.35 (63.69, 85)	58.8 (50.2, 68)	70.89 (60.57, 84)
Partial mediation	Yes	Yes	Yes	Yes	Yes
Full mediation	No	No	No	No	No
**Autonomy as mediator**					
Direct effect (ADE)	0.42 (0.33, 0.5)	0.26 (0.18, 0.34)	0.27 (0.18, 0.35)	−0.38 (−0.46, −0.3)	0.21 (0.12, 0.3)
Mediated effect (ACME)	0.43 (0.36, 0.51)	0.26 (0.18, 0.34)	0.4 (0.33, 0.47)	−0.43 (−0.5, −0.36)	0.41 (0.32, 0.5)
Mediation (%)	50.8 (43.7, 59)	49.8 (38.3, 61)	59.8 (50.5, 70)	53.3 (44.6, 62)	66.2 (53.7, 79)
Partial mediation	Yes	Yes	Yes	Yes	Yes
Full mediation	No	No	No	No	No

ADE, average direct effect; ACME, average causal mediated effect. Mediation (%) in bold means the mediation effect is “full mediation.”

### 3.5 Moderation analysis

**FIGURE 2:**
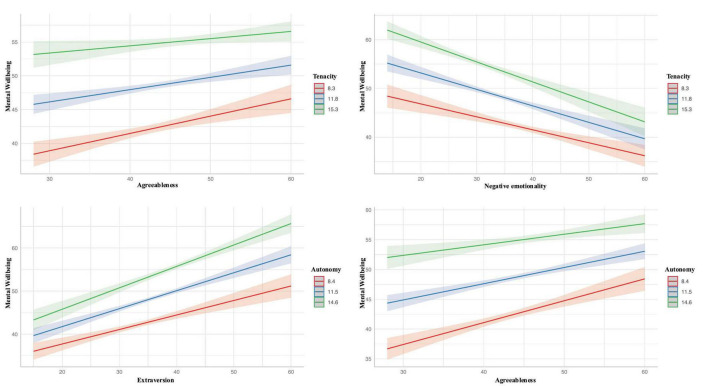
Moderation analysis results with resilience as moderator.

In the moderation analysis, most models did not reveal a significant conditional effect of self-esteem or personality traits on mental wellbeing, nor on resilience (*p* > 0.05). However, a significant interaction was found between the agreeableness personality trait and tenacity [β = −0.02 (−0.04, −0.001), *p* = 0.038], as well as between negative emotionality and tenacity [β = −0.02 (−0.04, −0.001), *p* = 0.008]. Additionally, autonomy as a moderating factor showed significant interactions with extraversion [β = 0.03 (0.007, 0.044), *p* = 0.005] and agreeableness [β = −0.03 (−0.054, −0.007), *p* = 0.011] personality traits. Figure 2 graphically illustrates these moderation effects, highlighting the role of resilience and its sub-dimensions as moderators.

## 4 Discussion

### 4.1 Summary of the findings

In this study, we aimed to investigate the intricate relationships between self-esteem, personality traits, resilience, and mental wellbeing among Chinese college students. Our findings shed light on the interconnected nature of these factors and contribute to our understanding of how they collectively impact mental wellbeing.

### 4.2 Sample character

In this cross-sectional study, 689 university students were recruited with the mean age 20.3 years. The paternal education and maternal education level of most of students is high school and below. Interestingly, this generational educational attainment suggests a potential for upward mobility among the participants. This observation raises the question of whether the participants are experiencing upward mobility, potentially influenced by their personality traits, self-esteem, and resilience. Additionally, this trend may mirror a societal progression in educational standards across China, indicative of national policy shifts and economic growth. It is crucial for future research to disentangle these individual and societal contributions to educational attainment and their subsequent impact on mental wellbeing.

### 4.3 Correlations

The findings of this study indicate a significant correlation between self-esteem, resilience, and mental wellbeing. Specifically, self-esteem was found to have a positive correlation with resilience and its three sub-dimensions. These findings align with earlier studies, underscoring the significant role of self-esteem, particularly relational self-esteem, in enhancing subjective wellbeing (Du et al., 2017). However, Liu et al. (2021) found that self-esteem had a negative relationship with mental wellbeing and a positive relationship with resilience, which contrasts with the findings of this study. Additionally, this study found significant correlations between personality traits, resilience, and mental wellbeing. Previous research has shown that certain personality traits are associated with resilience and mental wellbeing. For example, Oshio et al. (2018) found that high levels of neuroticism were associated with lower resilience and poorer mental wellbeing, while high levels of extraversion, conscientiousness, and agreeableness were associated with higher resilience and better mental wellbeing (Ercan, 2017). However, the relationship between self-esteem, personality traits, resilience, and mental wellbeing is complex and may be influenced by moderating or mediating factors (Waltz and Chou, 2022).

### 4.4 Mediation role of resilience

A key finding of this research is that the relationship between self-esteem and mental wellbeing is partially mediated by resilience and its three sub-dimensions. This can be interpreted to mean that individuals with high levels of self-esteem are more likely to have stronger resilience, which in turn can increase mental wellbeing. While few previous studies have investigated the mediating role of resilience, much research has focused on the mediating role of self-esteem (Bajaj et al., 2016). As such, direct comparisons are not available. However, studies have confirmed the mediating role of self-esteem in the relationship between psychological characteristics such as mindfulness and mental wellbeing outcomes such as stress (Bajaj, 2017). This may be the first study to investigate the mediating role of resilience in the relationship between self-esteem and mental wellbeing among Chinese college students. As such, this study expands our understanding of the mechanisms through which self-esteem impacts mental wellbeing.

In a review of research on resilience, Vella and Pai (2019) found that resilience is determined by complex interactions between different levels of systems, with individual traits being the most important factor at the top of the hierarchy. This means that individual traits have a vital influence on resilience. According to the socio-meter theory, self-esteem is one of these individual traits and is considered a motivational-affective system responsible for continuously monitoring a person’s social environment for signs of rejection and acceptance (Leary and Baumeister, 2000). Based on this theory and previous research, individuals with low self-esteem may experience a deterioration in their mental wellbeing through a pathway related to resilience. Envy may also play a role in the mediation effect of resilience. Individuals with high self-esteem may generate envy through social comparison, and previous studies among Chinese college students have shown that the association between envy and depression is mediated by resilience (Xiang et al., 2020). This study demonstrates that resilience partially mediates the impact of self-esteem on mental wellbeing, suggesting a new strategy for preventing mental wellbeing problems.

For the mediation role of resilience in the association between personality traits and mental wellbeing, resilience and adaptability fully mediated the impact of conscientiousness and open mindedness on mental wellbeing, while all the other mediation effect was partial and significant. One possible theory that could explain the findings is that resilience and adaptability act as mechanisms through which certain personality traits influence mental wellbeing (Oshio et al., 2018). Conscientiousness and open-mindedness may promote the development of resilience and adaptability, which in turn enhance mental wellbeing (Zager Kocjan et al., 2021). For other personality traits, the relationship with mental wellbeing may be more complex and involve additional factors beyond resilience and adaptability. Further research is needed to fully understand these relationships.

### 4.5 Moderation role of resilience

This study also examined the potential moderating effect of resilience on the relationship between self-esteem and mental wellbeing. Interestingly, the results showed that resilience did not have a significant moderating effect on this relationship. This can be interpreted to mean that the relationship between self-esteem and mental wellbeing is not significantly influenced (strengthened, weakened, or reversed) by resilience. In other words, regardless of an individual’s level or type of resilience, the relationship between self-esteem and mental wellbeing remains stable. This study appears to be the first to investigate the influence of resilience as a moderator in the relationship between self-esteem and mental wellbeing among Chinese college students. Although no moderation effect was found, this surprising finding still contributes to our understanding of the mechanisms through which self-esteem impacts mental wellbeing.

The study discovered that the relationship between agreeableness and mental wellbeing was moderated by autonomy and tenacity. In other words, individuals who scored high on agreeableness tended to have better mental wellbeing when they also possessed high levels of autonomy and tenacity, indicating that traits such as a sense of control and perseverance can enhance the inherently positive effects of agreeableness on mental wellbeing. Furthermore, the study found that tenacity also played a role in moderating the relationship between negative emotionality and mental wellbeing. Individuals who scored high on negative emotionality, but also exhibited high levels of tenacity, tended to have better mental wellbeing compared to those who lacked tenacity. On the other hand, autonomy was found to moderate the relationship between extraversion and mental wellbeing. Specifically, individuals who scored high on extraversion and also had high levels of autonomy tended to have better mental wellbeing. These findings highlight the importance of considering the roles of autonomy and tenacity in the relationships between personality traits and mental wellbeing.

The current findings align with existing literature that suggests personality traits, such as agreeableness, extraversion, and negative emotionality, can influence mental wellbeing (Markman, 2019). However, the moderating roles of autonomy and tenacity in these relationships are novel findings that contribute to the understanding of how these personality traits interact with personal characteristics related to autonomy and tenacity to impact mental wellbeing. This study provides further evidence that autonomy and tenacity are important factors that can potentially buffer the effects of certain personality traits on mental wellbeing, highlighting the complexity and multifaceted nature of the relationship between personality and mental health.

### 4.6 Limitations and strength

One significant limitation pertains to our study’s cross-sectional design. Employing this design restricts our ability to establish causality between variables. While our findings offer valuable insights into associations, they cannot definitively demonstrate the direction of influence. Future research with longitudinal approaches could provide stronger evidence in this regard.

Additionally, our study relied on self-report measures for data collection. While these measures are widely accepted and validated, they are inherently susceptible to response biases and subjectivity. Incorporating objective measures, such as physiological or behavioral data, could enhance the validity and reliability of our results.

For sampling, the absence of *a priori* sample size calculation, typical of exploratory pilot studies, means that our findings should be interpreted with caution, particularly when considering their statistical power and generalizability. Also, our sampling strategy, though designed to capture a diverse participant pool within Henan province, may not fully reflect the broader Chinese university student population. The study’s geographical focus on Henan, while offering a unique context due to its central location and mix of urban and rural settings, limits the extrapolation of our results to other regions in China with potentially different socio-cultural and educational environments.

Moreover, our analysis focused on the complex constructs of mediation and moderation involving resilience. While we aimed to provide comprehensive insights into these relationships, resilience is influenced by various individual and contextual factors that were beyond the scope of this study. Future investigations should consider these complexities.

Furthermore, our exploration of subjective wellbeing primarily targeted its cognitive aspects, such as life satisfaction and perceived quality of life, without delving into the affective components that encompass emotional reactions and mood states. This focus may limit our understanding of the full spectrum of subjective wellbeing, underscoring the need for future research to incorporate measures that assess both cognitive evaluations and affective experiences to ensure a comprehensive view of mental wellbeing.

While we hypothesized that stress reduction serves as a mediating factor from personality, and self-esteem to mental wellbeing, our study did not include direct measures of stress. This constitutes a limitation as stress levels can vary significantly among individuals and may influence the mediating effects observed. We recommend that future research include direct assessments of stress to provide more conclusive evidence on this pathway. This addition could offer deeper insights into the interplay between personality traits, self-esteem, and mental wellbeing.

Finally, it’s essential to recognize that, despite our efforts in data cleaning and validation, missing data may have influenced our results. This potential bias from missing data should be acknowledged and addressed in future studies.

On the other hand, our study boasts several strengths. We utilized a comprehensive battery of well-established scales to assess self-esteem, personality traits, resilience, and mental wellbeing. This thorough approach enhances the robustness of our findings and strengthens their reliability.

Furthermore, our research explored the mediation and moderation roles of resilience, contributing to a deeper understanding of the intricate mechanisms underlying the connections between self-esteem, personality, and mental wellbeing. These findings hold practical implications for interventions targeting mental health in young adults.

In conclusion, while our study acknowledges its limitations, such as the cross-sectional design, self-report measures, sampling method, and the complexities of resilience, it also offers valuable contributions to the study of self-esteem, personality traits, resilience, and mental wellbeing. These insights, supported by our comprehensive assessment and timely context, provide a valuable foundation for further research and interventions aimed at enhancing mental wellbeing in college students and beyond.

## 5 Conclusion

In conclusion, this study provides a comprehensive exploration of the intricate relationships between self-esteem, personality traits, resilience, and mental wellbeing among Chinese college students. The findings presented in this study offer valuable insights into the mechanisms underlying these relationships, enhancing our understanding of mental wellbeing dynamics.

The multifaceted nature of the interactions observed underscores the importance of considering a holistic perspective when addressing mental wellbeing challenges among college students. The results suggest that interventions aimed at fostering self-esteem, nurturing specific personality traits, and cultivating resilience could have a synergistic impact on promoting mental wellbeing. Moreover, the identification of the moderating effects of autonomy and tenacity on the personality-mental wellbeing relationship opens avenues for personalized intervention strategies.

While our study contributes to the growing body of literature in this field, several limitations warrant consideration. The cross-sectional design precludes establishing causal relationships, prompting the need for future longitudinal studies. Additionally, the reliance on self-reported measures introduces potential biases that warrant validation through diverse measurement approaches.

In practical terms, the implications of this study resonate beyond academia. Our findings offer guidance for the development of targeted interventions and programs aimed at bolstering mental wellbeing among college students, especially in times of adversity. These insights are particularly relevant given the ongoing challenges posed by the pandemic.

In closing, the present study lays a foundation for further exploration into the intricate web of factors shaping mental wellbeing. By deepening our understanding of the roles of self-esteem, personality traits, and resilience, we can pave the way for more effective strategies to enhance mental wellbeing and create a positive impact on the lives of college students.

## Data availability statement

The raw data supporting the conclusions of this article will be made available by the authors upon request.

## Ethics statement

The studies involving humans were approved by the Ethics Committee of Sias University, and the local schools that participated (project number: LL2022-0315). The studies were conducted in accordance with the local legislation and institutional requirement and with the Declaration of Helsinki. Online informed consent for participation in this study was provided by the participants.

## Author contributions

ZC: Conceptualization, Data curation, Formal analysis, Methodology, Project administration, Software, Writing – original draft, Writing – review & editing. ZL: Conceptualization, Data curation, Investigation, Software, Writing – original draft. JR: Data curation, Investigation, Software, Writing – review & editing. YC: Investigation, Software, Writing – review & editing. XT: Project administration, Supervision, Writing – review & editing.

## Footnotes

1
https://www.wjx.cn
